# Early-life stress impacts the developing hippocampus and primes seizure occurrence: cellular, molecular, and epigenetic mechanisms

**DOI:** 10.3389/fnmol.2014.00008

**Published:** 2014-02-10

**Authors:** Li-Tung Huang

**Affiliations:** ^1^Department of Pediatrics, Kaohsiung Chang Gung Memorial Hospital, Chang Gung University College of MedicineKaohsiung, Taiwan; ^2^Department of Traditional Chinese Medicine, Chang Gung UniversityLinkou, Taiwan

**Keywords:** early-life stress, epigenetic, epileptogenesis, hippocampus, hypothalamic-pituitary-adrenal axis, prenatal stress, postnatal stress, seizure

## Abstract

Early-life stress includes prenatal, postnatal, and adolescence stress. Early-life stress can affect the development of the hypothalamic-pituitary-adrenal (HPA) axis, and cause cellular and molecular changes in the developing hippocampus that can result in neurobehavioral changes later in life. Epidemiological data implicate stress as a cause of seizures in both children and adults. Emerging evidence indicates that both prenatal and postnatal stress can prime the developing brain for seizures and an increase in epileptogenesis. This article reviews the cellular and molecular changes encountered during prenatal and postnatal stress, and assesses the possible link between these changes and increases in seizure occurrence and epileptogenesis in the developing hippocampus. In addititon, the priming effect of prenatal and postnatal stress for seizures and epileptogenesis is discussed. Finally, the roles of epigenetic modifications in hippocampus and HPA axis programming, early-life stress, and epilepsy are discussed.

## Introduction

The early-life environment is one of the most important factors affecting life-long health (Anand, 2000; van den Bergh et al., 2005; Lupien et al., 2009; Boksa, 2010; Strüber et al., 2014). In humans, early-life stress is associated with a preterm birth and a low birth weight, and can prime the neonate for further complications later in life that include psychiatric disorders, aged-related cognitive dysfunction, obesity, and hypertension (Barker et al., 1989; Fowden et al., 2005; Lemaire et al., 2006; Lahiri et al., 2009; Strüber et al., 2014). Animal studies also suggest that exposure to stressors or steroids during early-life alter the programming of the hypothalamic-pituitary-adrenal (HPA) axis, neurobehavior, and neuroimmune systems (Matthews, 2000; Mueller and Bale, 2008; Lupien et al., 2009; Brunton and Russell, 2010; Chen and Zhang, 2011; Lai and Huang, 2011; Strüber et al., 2014). Epigenetic modification has gained increasing attention in recent years because of its connection with early-life adversities (Weaver et al., 2004; Meaney et al., 2007; Mueller and Bale, 2008; Chen and Zhang, 2011; McClelland et al., 2011a,b; Murgatroyd and Spengler, 2011; Lucassen et al., 2013; Rabbe and Spengler, 2013). On the other hand, stress during development can have a significant epigenetic impact on the brain, and this relationship is bidirectional (Hunter, 2012).

Early-life stressors include prenatal, postnatal, and adolescence stress (Lupien et al., 2009; Schmidt, 2010). For example, in humans, early-life stress can include prenatal stressors such as exposure to exogenous glucocorticoids, maternal infection (King et al., 2005; Sørensen et al., 2009; Jenkins, 2013), and birth complications, as well as postnatal stressors such as exposure to exogenous glucocorticoids, maternal postpartum depression, loss of a parent, exposure to family conflict and violence, neglect, or physical maltreatment (De Bellis, 2002; King et al., 2005; Frodl et al., 2010). Both prenatal and postnatal stress can increase the likelihood of seizures in early life (Joels, 2009; Koe et al., 2009) and epileptogenesis in later life. This article focuses only on the influences of prenatal stress and postnatal stress.

## Hippocampal and HPA axis development

The hippocampus develops primarily during the fetal period in both rodents and primates (Seress et al., 2001; Khalaf-Nazzal and Francis, 2013). The limbic system, which includes the hippocampus, amygdala, and anterior cingulate cortex are already formed during the third and fourth month. Dentate gyrus forms at late stages of embryogenesis, however small numbers of dentate gyrus cells are formed from mid-embyrogenesis making temporal matching and connectivity of cells from other hippocampal subfields (Deguchi et al., 2011). Rodents and primates differ in the timing at which the majority of the dentate granule cells are produced; however, both rodents and primates produce ~85% postnatally (Bayer, 1980a; Rakic and Nowakowski, 1981). A similar percentage of cornus ammonis (CA) 1–3 subfield neurons are produced during the last days of gestation in rodents, and during the first half of pregnancy in primates (Bayer, 1980b; Rakic and Nowakowski, 1981). The hippocampal subfields can be recognized with distinct molecular markers from embryonic stages (Khalaf-Nazzal and Francis, 2013).

In the rodent, maturation and full differentiation of the hippocampal formation takes place during early postnatal periods (Avishai-Eliner et al., 2002). During the first postnatal weeks, neuronal birth, differentiation, and migration are ongoing (Altman and Bayer, 1990; Gould and Cameron, 1996). Neurogenesis of granule cells peaks during the second week of life in rodents (Bayer, 1980a), and during the third month in humans (Seress et al., 2001). In addition, synaptogenesis and the establishment of enduring connectivity patterns continue for weeks in the rodent, and for years in humans (Avishai-Eliner et al., 2002).

Glucocorticoids are released from the adrenal glands in response to stress, readily cross the blood-brain barrier, and activate hippocampal glucocorticoids receptors (McEwen, 1998). Glucocorticoids interact with their receptors in multiple target tissues, especially the HPA axis. Glucocorticoids act via two intracellular receptors, the glucocorticoid receptor (GR) and the mineralocorticoid receptor (MR) to regulate gene transcription. In addition, glucocorticoids can change neural function via rapid nongenomic actions. GR and MR differ in ligand affinity and distribution (de Kloet et al., 2005): GR has a lower affinity than MR has, and therefore are more frequently occupied when corticosterone levels increase (de Kloet et al., 2005). The actions of glucocorticoids depend on the functionality of the balance between GR and MR in the brain (de Kloet et al., 2005).

There is a distinct ontogenic profile for GR and MR in the fetal rat brain (Diaz et al., 1998). GR mRNA is present in the anterior hypothalamus, hippocampus, and pituitary by gestational day 13 (Diaz et al., 1998), whereas MR mRNA is present in the hippocampus by gestational day 16 and the hypothalamus by day 17 (Diaz et al., 1998). GR and MR in the rat fetal brain are low throughout gestation, but increase rapidly after birth, consistent with the postnatal development of the brain in the rat (Diaz et al., 1998).

During pregnancy, the mother's HPA axis undergoes major changes (Lindsay and Nieman, 2005). Cortisol secretion increases steadily through gestation (Jung et al., 2011); thus, the normal physiological responses to stressors and the cortisol awakening response (i.e., basal HPA activity) are attenuated (Lindsay and Nieman, 2005). For most of the pregnancy, the baby and mother share a common corticotrophin-releasing hormone (CRH)-adrenocorticotropic hormone (ACTH)-cortisol axis (McLean et al., 1995).

By the end of the first week of life (Bohn et al., 1994; Vazquez et al., 1998), the number of MRs reaches adult levels. The number of GRs present during the first few week of life, however, is ~30% of adult levels, but approach adult levels after ~30 days of life. Both GR and MR are highly expressed in the developing brain, and have different and complex ontogenies that allow intricate brain development.

Between postnatal day 4 and 14, neonatal rat pups have low basal corticosterone levels and the corticosterone response to stressors is blunted, which constitutes the so-called stress hyporesponsive period (SHRP) (Levine, 2005). However, disruption of normal maternal behavior in rat during the SHRP can influence HPA axis development. In humans, the HPA axis is highly reactive and labile during early infancy, but organizes between 2 and 6 months of age through interactions between the infant and caregiver. The quality of caregiving that the infant receives predicts the infant's ability to self-regulate later in life. Sensitive caregiving is associated with better self-regulatory abilities and optimal functioning of the child's HPA system (Gunnar and Cheatham, 2002; Gunnar and Donzella, 2002).

## Effects of pre-/post-natal stress on seizure susceptibility and epileptogenesis

Epileptogenesis is a process through which the normal brain develops epilepsy, and the hippocampus is implicated in the pathogenesis of both the initiation and propagation phases (Pitkänen and Lukasiuk, 2011). Mesial temporal lobe epilepsy (MTLE), the most common focal intractable epilepsy, is thought to be a multi-stage process of increasing epileptogenesis commencing in early life. The ongoing process of epileptogenesis and the course of epilepsy might be negatively influenced by the stress associated with the disease itself (Joels, 2009; Sawyer and Escayg, 2010). As a result, a negative loop might occur in which stress promotes epileptogenesis in predisposed individuals or lowers seizure threshold in epilepsy patients, thereby increasing the likelihood of exposure to stress, which in turn exacerbates the disease. Epidemiological data implicate stress in the cause of epilepsy and seizures in both children and adults (Temkin and Davis, 1984; Swinkels et al., 1998; Bosnjak et al., 2002).

Stress is a natural factor that may exacerbate or trigger seizures (Novakova et al., 2013; van Campen et al., 2013). HPA-related stress hormones, especially glucocorticoid and CRH, can affect excitatory and inhibitory processes in brain areas that are critically involved in seizure generation. Glucocorticoid exposure can alter plasticity in the hippocampus through increasing extracellular glutamate levels and calcium conductance (either voltage- or ligand-gated), alter expression of N-methyl-D-aspartate (NMDA) receptor subunits, and reduce glial uptake of glutamate, and thus, facilitate epileptiform discharges and seizures in animals. Glucocorticoids facilitate epileptiform discharges and seizures in animals. CRH is expressed in interneurons in both the developing and adult hippocampus and is released during stress (Sakanaka et al., 1987; Chen et al., 2001). Both glucocorticoids and CRH are important hormones that regulate the stress response and may contribute to seizure-induced loss of neurons, dendritic spines, and branching if it persists for a prolonged period (Ribak and Baram, 1996; Chen et al., 2012).

Negative life events and stress sensitivity are linked with childhood epilepsy (van Campen et al., 2012, 2013). In addition, epidemiological data implicate stress in the causation of epilepsy and seizures in children (Bosnjak et al., 2002). Specifically, early-life stress might create an enduring vulnerability to limbic epilepsy through altering glucocorticoids (Kumar et al., 2007), HPA axis (Joels, 2009), CRH (Baram and Hatalski, 1998), inflammation (Vezzani et al., 2013), membrane receptors such as gamma-aminobutyric acid (GABA) (Reddy, 2013), NMDA (Olney et al., 1991), and 2-amino-3-(3-hydroxy-5-methylisoxazol-4-yl) propionic acid (AMPA) receptors and neurotransmission (Rogawski, 2013), cellular electrophysiology, such as long-term potentiation (LTP) and long-term depression (Blaise et al., 2008), limbic area structures (Wong and Guo, 2013), and neuronal cell proliferation and neurogenesis (McCabe et al., 2001).

## Physiological mechanisms by which pre-/post-natal stress affects the developing hippocampus

### Prenatal stress

#### Glucocorticoid hormones

During pregnancy, women have naturally elevated levels of cortisol. In general, normal glucocorticoid concentrations are essential for the development of several organs, including the central nervous system. Prenatal stress or synthetic glucocorticoid administration exposes the fetus to high glucocorticoid levels, which leads to downregulation of GR in the hippocampus, attenuation of negative feedback for the HPA axis, and enhanced HPA axis activity (Reul and de Kloet, 1985; Harris and Seckl, 2011).

#### Placental CRH

In humans, placental CRH activity is modulated by the maternal HPA axis (Wadhwa et al., 1998). Placental CRH concentration is a significant predictor of spontaneous preterm birth (Glynn et al., 2001; Sandman et al., 2006) and intrauterine growth restriction (IUGR) (Wadhwa et al., 2004), and can influence hippocampal development in the fetus. Prenatal stress activates the maternal HPA axis, which increases placental CRH production and its subsequent release into the bloodstream. A positive feed-forward loop between cortisol and placental CRH indicates that prenatal stress leads to progressively higher fetal plasma CRH levels. Placental CRH may penetrate the blood-brain barrier of the fetus, and subsequently influence both the function and the integrity of the hippocampus (Kastin and Akerstrom, 2002), presumably by activating CRH receptors (Sandman et al., 1999; Wadhwa et al., 2001).

#### Placental 11β-hydroxysteroid dehydrogenase type 2 (11β-HSD2)

The placenta is an effective barrier between the maternal and fetal hormonal environments in humans, being rich in 11β-HSD2, which converts cortisol to inactive cortisone (Benediktsson et al., 1997). Downregulation of placental 11β-HSD 2 increases glucocorticoid exposure for the placenta and fetus. Maternal stress not only increases her own circulating cortisol, it also reduces the expression and activity of 11β-HSD 2 in the placenta, leaving the fetus less protected (Avishai-Eliner et al., 2002; Mairesse et al., 2007). Moreover, inhibition of 11β-HSD2 might contribute to low birth weight, IUGR, and pregnancy disorders such as preterm birth and preeclampsia (Causevic and Mohaupt, 2007; Michael and Papageorghiou, 2008).

#### Impaired uterine blood flow

The impact of maternal anxiety on fetal blood flow can be determined by using ultrasound to measure the blood flow pattern in the uterine arteries. Sjostrom et al. found that, at 37–40 gestational weeks, mothers with high-trait anxiety scores had fetuses with higher indices of blood flow in the umbilical artery, and lower values in the fetal middle cerebral artery, suggesting a change in blood distribution that favored brain circulation (Sjöström et al., 1997).

### Postnatal stress

#### CRH

CRH is expressed in hippocampal interneurons and is released from axon terminals during stress. CRH is produced in several populations of cells in the developing hippocampus, such as Cajal-Retzius cells, and is involved in the maturation of hippocampal circuitry (Chen et al., 2001).

Chronic early-life stress, which was imposed by creating “simulated poverty” in the cage, resulted in cognitive problems and dendritic atrophy with loss of dendritic spines and synapses (Brunson et al., 2005). Many of the persistent effects of early-life stress are reversible with subsequent treatment with a CRH receptor 1 (CRHR_1_) antagonist (Fenoglio et al., 2005). Adult mice lacking CRHR_1_ in the forebrain were relatively resistant to the deleterious effects of chronic stress of social defeat (Wang et al., 2011a). Interestingly, the local deletion of CRHR_1_ also protected adult mice from the adverse effects of chronic early-life stress on learning and memory (Wang et al., 2011b). Infusion of CRHR_1_ antagonists immediately following this early-life stress prevented the learning and memory deficits, rescued LTP, and restored the integrity of the dendritic structure (Ivy et al., 2010). These findings provide direct evidence for a need for CRH-CRHR_1_ signaling in the persistent effects of chronic early-life stress on hippocampal synapses. In this regard, Karsten and Baram propose that early-life experience can result in persistently altered regulation of CRH expression, which provides the neurobiological substrate to subsequent stress and some adult psychopathology (Karsten and Baram, 2013). In line with the preclinical data, single-nucleotide polymorphisms in the CRHR_1_ gene protect against depression in individuals exposed to childhood maltreatment (Tyrka et al., 2009).

#### Glucocorticoid hormones

Glucocorticoids are released from the adrenal glands in response to stress, readily cross the blood-brain barrier, and activate hippocampal glucocorticoids receptors (McEwen, 1998). Schmidt et al. demonstrated that glucocorticoid excess during the SHRP has only limited consequences on the adult behavioral phenotype (Schmidt et al., 2002). In addition, glucocorticoid administration early in life does not reproduce the effects of stress on hippocampal function and integrity when given in a non-stressful manner (Leverenz et al., 1999). Together, glucocorticoids play a minor role, and other factors may contribute more to the mechanisms by which early-life stress influences hippocampal development and function throughout life.

## Prenatal stress

Prenatal stress is an important programming factor in brain development and function. A recent cross-sectional study indicated that 6% of pregnant women reported high levels of psychological stress during their pregnancies that resulted from conditions including depression, panic disorder, or domestic violence (Woods et al., 2009). Talge et al. reviewed several prospective studies related to prenatal maternal stress, and found a substantial number of emotional/behavioral problems in children, including attention deficit hyperactivity disorder, anxiety, and language delay, that were attributed to prenatal stress or anxiety in ~15% of the subjects (Talge et al., 2007).

## Cellular and molecular alterations in the developing hippocampus that may link prenatal stress to seizure and epileptogenesis

### Glucocorticoid and CRH and HPA axis

The density of hippocampal GRs was lower by ~50% in prenatal stress female offspring; however, no difference was observed between prenatally stressed and control males (Szuran et al., 2000). This female-specific decrease in hippocampal GRs was also shown by Weinstock et al. (1992).

Szuran et al. restrained pregnant rat dams for 30 min/day during gestational days 15–19. Prenatally stressed females had higher basal corticosterone levels (Szuran et al., 2000). Exposure to exogenous glucocorticoids during the last week of gestation increased basal and stress-induced plasma corticosterone levels in adult rats (Seckl, 2004) and attenuated the HPA axis response (Seckl, 2004; Welberg and Seckl, 2001). Endogenous glucocorticoids mediated some of the changes in HPA responsiveness in prenatally stressed offspring, both in rodents and primates (Matthews, 2000).

### Inflammation

Restrained pregnant mice dam offspring showed increased interleukin-1β and tumor necrosis factor-α level in the hippocampus, increased interleukin-1β immunoreactive microglial cells, and increased activated microglia. In addition, systemic administration of lipopolysaccharide induced a significant increase in tumor necrosis factor-α in the hippocampus of only prenatally stressed mice but not non-stressed animals (Diz-Chaves et al., 2012, 2013).

### Membrane receptors and neurotransmitter

Maternal immune activation caused reduced basal neurotransmission of dopamine and glutamate, as well as reduced levels of the inhibitory transmitter GABA, within the hippocampus (Bitanihirwe et al., 2010). Prenatal stress also reduced the expression and activity of metabotropic glutamate receptor 5, which is implicated in the regulation of synaptic plasticity and neurogenesis in the hippocampus of male rats (Morley-Fletcher et al., 2011).

### Cellular electrophysiology

A significant downregulation of hippocampal genes also was reported in 23-day-old female rats whose mothers were stressed from gestational days 17–21 (Bogoch et al., 2007). This included presynaptic voltage-gated Ca^2+^ type P/Q and several K^+^ channels that regulate the neuron membrane potential and suggests a potential decrease in the excitability of newly formed synapses.

### Spine and dendrite and cell morphology

Hayashi and colleagues reported that rats exposed to prenatal stress had a significant 32% reduction in synaptic density within the hippocampal CA3 area, as measured on postnatal day 35 (Hayashi et al., 1998). Lemaire et al. (2000) reported a reduction in the number of granule cells within the hippocampal dentate gyrus of prenatally stressed rats measured 28 days postnatally.

### Neuronal cell proliferation and neurogenesis

In male mice, prolonged prenatal stress decreased cell proliferation in the hippocampus by 60% on postnatal day 10 (Kawamura et al., 2006). In another experimental paradigm, daily maternal restraint during the last week of gestation resulted in deficits of hippocampal neurogenesis (Lemaire et al., 2006). The relationship between prenatal stress and neurogenesis is complicated and depends on the stressor type, sex, and environment. Prenatal stress seems to have both enhancing and suppressing effects on the development of hippocampal neurons in a stressor intensity-dependent manner (Fujioka et al., 2006). Fujioka et al. reported that short-lasting (i.e., 30 min, once daily, between gestation days 15–17) and mild prenatal stress seemed to enhance neonatal neurogenesis, facilitate LTP, and the differentiation of processes of hippocampal neurons, whereas long-lasting (i.e., 240 min, once daily, between gestation days 15–17) and severe prenatal stress impaired their morphology.

## Effects of prenatal stress on seizure susceptibility and epileptogenesis

Beck and Gavin treated pregnant mice with beta-2-thienylalanine solvent or a sham injection on gestational days 10–12. Audiogenic seizures were tested on postnatal day 23. An increase in audiogenic seizure frequencies were observed in injected mice, irrespective of the nature of the injected substance. This finding suggested that the act of manipulation, rather than the test substance, caused stress and increased seizure propensity (Beck and Gavin, 1976). Frye and Bayon exposed rats to 20 min of restraint stress toward the end of their pregnancy (Frye and Bayon, 1999). They found that the prenatally stressed offspring had more partial seizures and tonic-clonic seizures with long durations than did control rats. Edwards et al. examined how stress exposure at different times during gestation might affect later limbic system excitability and the propensity to develop epilepsy (Edwards et al., 2002). Pregnant dams were restrained under bright light for 45 min, three times a day during either early gestation (gestational days 5–12) or mid-late gestation (gestational days 12–20). Offspring of the stressed dams were then tested as an infant at postnatal day 10 or as adults, and were compared with offspring from non-stressed dams. Outcome measures assessed were the stimulation-induced seizure threshold, after-discharge threshold, and the rate of seizure development using electrical hippocampal kindling. Both prenatal stressors significantly lowered after-discharge threshold in pups, but this effect appeared to diminish by adulthood in the early gestational stress group. In addition, mid to late gestational stress accelerated kindling rates in all infant offspring and in adult males, but had no effect in adult female rats. Notably, Young et al. administered dexamethasone or betamethasone on gestational days 15–18, and tested the seizure threshold and kindling parameters (Young et al., 2006). They found prenatal betamethasone treatment increased seizure threshold for both models. Prenatal dexamethasone treatment increased kindling threshold, but not seizure threshold. Kindling rate was unaffected by either glucocorticoid treatment (Young et al., 2006). Velisek showed prenatal exposure to betamethasone decreased postnatal susceptibility to flurothyl-induced clonic seizures but not to kainic acid-induced seizures. Prenatal hydrocortisone decreased postnatal weight but did not affect seizure susceptibility (Velíšek, 2011). In their subsequent work, Yum et al. demonstrated that prenatal restraint stress (2 × 45 min) in rats on gestational day 15 would increase susceptibility to spasms on postnatal day 15 (Yum et al., 2012).

Shang et al. showed an association between the onset risk of infantile spasms and the degree of maternal stress (Shang et al., 2010). However, in a population-based cohort study in Denmark, Li et al. studied children who were hospitalized because of epilepsy and born to women who had lost a close relative during pregnancy 1 year before pregnancy (Li et al., 2008). In this study, no association was found between this particular form of prenatal stress and the risk of epilepsy.

Indirect evidence links prenatal stress and an increased likelihood of childhood seizures in children with autistic disorder. Minshew et al. pointed out that epilepsy is found in about one-third of patients with autistic disorder, a disorder related to prenatal stress (Kinney et al., 2008), compared with a prevalence of only 2–3% in the general population (Minshew et al., 2005). Table 1 summarizes the current rodent studies regarding the impact of prenatal stress on seizure occurrence and epileptogenesis.

**Table 1:** **Summary of rodent studies investigating effects of prenatal stress in rodent models of epilepsy/epileptogenesis**.

**Author**	**Manipulation in prenatal life**	**Endpoint test of seizure threshold or epileptogenesis**	**Outcome measurements**	**Conclusions/implications**
Beck and Gavin, 1976	Pregnant dams received beta-2-theinylalanine or solvent on GDs 10–12 Control: unhandled mice	Audiogenic seizures on PND 23	Increased seizure frequencies in injected mice, irrespective of the nature of the injected substance	Prenatal stress increased seizure susceptibility in young age
Frye and Bayon, 1999	Maternal restraint stress of mother for 20 min on GD 18 Control: no restraint stress rats	Adult gonadectomized offspring were administered 3 alpha, 5 alpha-THP 1h prior to testing for kainic acid-induced seizures	Increased seizure production and longer duration in stressed offspring Lower dose of 3 alpha, 5 alpha-THP was effective in reducing seizure duration in control females Higher dose of 3 alpha, 5 alpha-THP was needed to reduce seizure duration in prenatally stressed females and males	Prenatal stress decreases neurosteroid's anti-seizure capability. Effects are sex-dependent
Edwards et al., 2002	Midde restraint stress (45 min, 3×/day, GDs 5–12) Late restraint stress (45 min, 3×/day, GDs 12–20)	ADT and Hippocampus kindling on PND 14 or in adults	Lowered ADT on PND 14 infant rat offspring in both early and late gestation stressed rats. Increased kindling rate in infant and adult male offsprings of middle and late gestation stress, but not in females. No effect on ADT	Prenatal stress, in particular during the latter half of gestation, increases seizure vulnerability in the unborn offspring. The offspring appear most susceptible to seizure development during the infantile period, but some effects persist into adulthood, particularly in males
Young et al., 2006	Pregnant dams received once daily injections with dexamethasone (0.2 mg/kg/day) or betamethasone (0.2 mg/kg/day) between GDs 15–18	Seizure thresholds were determined on PND 14 using electroconvulsive shock. Hippocampus kindling on PNDs 14–15	Prenatal betamethasone increased seizure threshold for both models. Prenatal dexamethasone increased kindling threshold, but not electroconvulsive shock threshold. Kindling rate was unaffected by either prenatal glucocorticoid	Prenatal repeated glucocorticoid treatments raised seizure thresholds and reduced seizure vulnerability, seemingly “favorable”
Velíšek, 2011	Pregnant dams received hydrocortisone (2 × 10mg/kg) or betamethasone (2 × 0.4 mg/kg) on GD 15	Seizures induced by flurothyl or kainic acid on PND 15	Prenatal exposure to betamethasone decreased postnatal susceptibility to flurothyl-induced clonic seizures but not to kainic acid-induced seizures. Prenatal hydrocortisone did not affect seizure susceptibility	Prenatal exposure to glucocorticoids on seizure susceptibility may be seizure syndrome specific
Yum et al., 2012	Prenatal restraint stress (2 × 45 min) GD 15	Development-specific spasms triggered by NMDA on PND 15	Prenatal stress significantly accelerated onset and increased number of NMDA-triggered spasms	Prenatal stress may enhance susceptibility to develop triggered spasms in infant rats. This finding is similar to increased risk for development of infantile spasms in children of mothers with gestational stress

ADT, afterdischarge threshold; GD, gestational day; NMDA, N-methyl-Daspartate 3 alpha; PND, postnatal day; 5 alpha-THP, 5 alpha pregnan-3 alpha-ol-20-one.

## Postnatal stress

Early-life adversity (childhood abuse and neglect, loss of parents, or extreme poverty) occurs worldwide and are all too common in the lives of children (Jones, 2008; Sandberg and Rutter, 2008). In the Dunedin Study birth cohort of 1037 children, followed prospectively for 32 years, maltreatment includes maternal rejection, harsh discipline, sexual abuse, physical abuse, and disruptive caregiver changes (Danese et al., 2009). For each child, the cumulative index counts the number of maltreatment indicators experienced during the first decade of life; 63.7% of children experienced no maltreatment, 26.7% experienced one form of maltreatment, and 9.6% experienced two or more forms of maltreatment (Danese et al., 2009). Clinical evidence from life-course epidemiology study points to the importance of early life experiences in shaping adult disease (Poulton et al., 2010).

## Cellular and molecular alterations in the developing hippocampus that may link postnatal stress to seizure and epileptogenesis

### Glucocorticoid and CRH and HPA axis

A 24-h maternal separation paradigm in 11-day-old rat pups can lead to a decrease in the expression of GR and MR mRNA in the hippocampus (van Oers et al., 1998). Likewise, expression levels of GR and MR are down regulated in the hippocampus of maternally separated mice on postnatal day 9 (Schmidt et al., 2002). In addition, neonatal infection in mice led to altered hippocampal GR and MR mRNA, as well as proteins, following a subsequent adult infection (Wynne et al., 2011).

Wang et al. demonstrated that early postnatal life stress impairs hippocampus-dependent spatial learning and memory in adult mice, and is associated with physiological, morphological, and molecular abnormalities in the hippocampus (Wang et al., 2011a,b). Impairments of spatial learning and memory in early postnatal life stress are recapitulated by forebrain CRH overexpression and attenuated by forebrain CRHR_1_ inactivation. This suggests the forebrain CRH-CRHR_1_ system is crucial for modulating and programming cognitive functions by early-life stress (Wang et al., 2011a,b).

### Inflammation

In rat, maternal separation on postnatal day 9 caused increased hippocampal interleukin-1 receptor in male offspring (Viviani et al., 2014). In the hippocampus, a decrease in BDNF mRNA and an increase in interleukin-1β mRNA were observed in rats with a neonatal infection and an immune challenge in adults (Bilbo et al., 2008).

### Membrane receptors and neurotransmitter

Maternal separation on postnatal day 9 decreased the levels of the AMPA receptor GluA1 and GluA2 subunits, altered NMDA receptor subunits GluN2B to GluN2A ratio, and increased interleukin-1 receptor interactions with GluN2B at the synapse of male hippocampal neurons (Viviani et al., 2014). This mechanism is part of a complex re-organization of the excitatory glutamatargic synapses. Hsu et al. reported two episodes of handling with maternal separation during early postnatal development resulted in long-term changes in postsynaptic GABA receptor function and subunit expression in hippocampal dentate gyrus (Hsu et al., 2003).

### Cellular electrophysiology

Maternal separation prevented the stress-induced transformation from early to late LTP in the dentate gyrus of adult male rats (Wang et al., 2013b). However, maternal separation for 24h on postnatal day 3 facilitated LTP in the dentate gyrus after an acute stress (Oomen et al., 2010).

### Spine and dendrite and cell morphology

An altered granule cell dendritic morphology (Oomen et al., 2010), a lower number of hippocampal neurons and glia (Leventopoulos et al., 2007; Fabricius et al., 2008), and a reduced mossy fiber density (Hout et al., 2002) have been reported following maternal separation (Rodenas-Ruano et al., 2012). Wang et al. demonstrated that postnatally stressed adult mice had decreased hippocampal nectin-3 levels and dendritic spine loss via CRH mechanism (Wang et al., 2013a).

### Neuronal cell proliferation and neurogenesis

Maternal separation for 180 min leads to an increase in cell proliferation on postnatal day 21 (Nair et al., 2007); however, in 2- to 7-month-old rats, cell proliferation was reduced (Mirescu et al., 2004; Oomen et al., 2010; Hulshof et al., 2011).

Maternal separation for 24h on postnatal day 3 increases hippocampal neurogenesis (Oomen et al., 2009). Similar to cell proliferation, early stress is associated with distinct consequences on hippocampal neurogenesis that manifest in a temporally regulated manner, i.e., enhanced in young adulthood and impaired in middle-aged (Suri et al., 2013).

## Effects of postnatal stress on seizure susceptibility and epileptogenesis

Edwards et al. investigated the effects of maternal separation on kindling epileptogenesis utilizing a relatively benign separation protocol that included 60 min on postnatal days 4 and 5 (Edwards et al., 2002). The comparison group included the other littermates, which were briefly handled but not removed from the mother. This postnatal manipulation had no effect on after-discharge threshold or rapid hippocampal kindling rates when assessed at 2 weeks of age.

To investigate the effects of maternal separation on the long-term consequences of early-life status epilepticus, Lai et al. tested whether maternal separation for 1h affected the long-term sequelae of emotional disorders following seizure early in life (Lai et al., 2006). Lai et al. used maternal separation that involved 1h of isolation daily during postnatal days 2 and 9, and used lithium-pilocarpine-induced status epilepticus on postnatal day 10 rats. As adults, anxiety-related behavior was assessed using the elevated plus maze test and seizure susceptibility was assessed by pentylenetetrazol-induced seizures. Rats exposed to maternal separation and seizures demonstrated a reduced pentylenetetrazol threshold for seizure induction compared to non-handled rats or rats exposed to isolation or seizure alone. Metyrapone (a corticosterone synthesis inhibitor) treatment prior to seizure did not reverse this enhanced excitability, indicating a partial role of glucocorticoids in this context. Salzberg et al. examined the effects of maternal separation on limbic excitability and the development of amygdala kindling (Salzberg et al., 2007). Postnatal stress was induced by separating pups from their mothers for 180 min daily from postnatal days 2–14. The comparison condition was mother and pup separation for 15 min per day over the same period, an exposure referred to as early handling. At 8 weeks of age, equivalent to young adult life, rats were tested for the after-discharge threshold and subjected to rapid amygdala kindling. Rats exposed to early-life stress exhibited significantly lower seizure thresholds and an accelerated rate of kindling, compared to early handled rats. These effects on limbic excitability and epileptogenesis were specifically observed in female rats, whereas males did not demonstrate changes in epilepsy outcomes, despite demonstrating increases in anxiety-like behavior. Using the rat amygdala-kindling model, Kumar et al. demonstrated that early-life stress induced by maternal separation accelerates the progression of focal limbic seizures to secondary generalized convulsive seizures in adult rats (Kumar et al., 2011). Desgent et al. used a two-hit model of TLE characterized by two early-life insults: a freeze lesion-induced cortical malformation on postnatal day 1, and a prolonged hyperthermic seizure on postnatal day 10 (Desgent et al., 2012). They demonstrated that after both insults, females did not develop MTLE while all males did. This correlated with a rise in corticosterone levels on postnatal day 1 following the lesion, but only in males. Their data demonstrated sexual dimorphism in the long-term vulnerability for developing epilepsy in the lesion plus hyperthermia animal model of MTLE, and suggested that the response to early-life stress at postnatal day 1 contributed significantly to epileptogenesis in a sex-specific manner (Desgent et al., 2012). Ali et al. demonstrated changes in firing patterns in thalamocortical and hippocampal regions resulting from both maternal separation and amygdala kindling, which might reflect cellular changes underlying the enhanced vulnerability to kindling in rats that had been exposed to early-life stress (Ali et al., 2013).

Similarly, Leussis and Heinrichs cross-fostered El pups to CD-1 dams because CD-1 dams exhibit a higher quality of maternal care than El dams. El pups raised by CD-1 dams experienced delayed seizure onset and reduced seizure frequency, suggesting that early-life environment can play an important role in shaping the adult seizure phenotype (Leussis and Heinrichs, 2009). It should be noted the El mouse model has not been verified for its effect on early-life stress. In addition, El pups raised in a biparental environment with both the El dam and sire attending the pups received more parental attention than El pups raised by only the El dam, yet they showed an earlier development of seizures (Orefice and Heinrichs, 2008). Together, early-life environment can interact with a genetic predisposition to shape the future seizure phenotype.

Van Campen et al. studied the effect of stress on seizure frequency in childhood epilepsy. They found stress sensitivity was reported in half of the children with epilepsy. They suggested that experiencing negative life events might cause a larger response to daily stressors, thereby increasing the likelihood to induce epileptic activity in childhood (van Campen et al., 2012). Table 2 summarizes the current rodent studies regarding the impacts of postnatal stress upon seizure occurrence and epileptogenesis.

**Table 2:** **Summary of rodent studies investigating effects of postnatal stress in rodent models of epilepsy/epileptogenesis**.

**Author**	**Manipulation in postnatal life**	**Endpoint test of seizure threshold or epileptogenesis**	**Outcome measurements**	**Conclusions/implications**
Edwards et al., 2002	Maternal separation (1 h/day, PNDs 4–5) Control: non-stressed littermates	ADT and Hippocampus kindling on PND 14	No effect on ADT or kindling rate	Postnatal stress has no effect on infant seizure susceptibility
Lai et al., 2006	Maternal separation (1 h/day, PNDs 2–9) and SE induced by lithium-pilocarpine Control: normal rearing and SE induced by lithium-pilocarpine	pentylenetetrazole-induced seizures at PND 100	Prolonged seizure duration and reduced seizure threshold following early life SE in stressed rats	Early life stress increases the vulnerability to seizures in adulthood
Salzberg et al., 2007	Maternal separation (180 min/day, PNDs 2–14) Control: EH (separation 15 min/day, PNDs 2–14)	Rapid amygdala kindling on ~PND 56	Stress female rats had increased kindling rate and reduced seizure threshold; no differences in male	Early life stress contributes to epileptogenesis. Effects are sex-dependent
Orefice and Heinrichs, 2008	Amount of parental care between PNDs 2–21 on genetically susceptible El mouse seizure emergence	HISS test on PNDs 80–90	HISS testing of adult El offspring revealed a deleterious effect of biparental rearing as a second care provider is a stressor in El pups	Early life stress increased seizure suscebtibility in adult El mice
Leussis and Heinrichs, 2009	Cross-fostering genetically susceptible El pups to a seizure-resistant CD-1 mothers	HISS test on PNDs 80–90	cfos hypoactivity in hippocampus and cortex on PNDs 35–40 as a result of HISS. El mice offspring with improved maternal care showed delayed onset of HISS-induced seizure susceptibility on PNDs 80–90	Increased maternal care in genetically susceptible El mouse may have prophylactic benefits for neural plasticity and adult seizure susceptibility
Kumar et al., 2011	Maternal separation (180 min/day, PNDs 2–14) Control: EH (separation 15 min/day, PNDs 2–14)	Rapid amygdala kindling on ~PND 56	Stress rats has accelerating kindling rates, enhanced corticosterone response to kindled seizure, decreased hippocampal pyramidal cell numbers, and enhanced kindling-induced neurogenesis in adulthood	Alternations of hippocampal pyramidal cell neurogenesis are candidate mechanisms that early life stress promotes vulnerability to epileptogenesis. Effects are sex dependent
Desgent et al., 2012	Two early life insults: a freeze lesion-induced cortical malformation at PND 1 and a hyperthermic seizure at PND 10	Video-EEG from PND 90 to 120	Increased susceptibility to PND 10 hyperthermia-induced convulsion in PND 1 lesioned rat. Two hits in females did not develop mesial temporal lobe epilepsy while all males did	Early life stress contributes to epileptogenesis. Effects are sex-dependent
Ali et al., 2013	Maternal separation (180 min/day, PNDs 2–14) Control: EH (separation 15 min/day, PNDs 2–14)	Amygdala kindling	Hippocampus: stress rats had more % APs firing in burst	Sress rats had enduring alterations in the firing patterns of neurons in the hippocampus that may underlie the increased vulnerability to limbic epileptogenesis

ADT, afterdischarge threshold; APs, action potentils; EEG, electroencephalogram; EH, early handling; HISS, handling-induced seizure susceptibility; PND, postnatal day; SE, status epilepticus.

## Epigenetic modifications in development programming and the effects of stress

Epigenetic modifications regulate gene expression without altering the DNA sequence. Epigenetic changes involve DNA methylation at cytosine-guanine sequences-CpG sites, histone posttranslational modifications (histone methylation, acetylation, phosphorylation, ubiquitylation, sumoylation, and propionylation), and microRNAs (Gräff et al., 2011). Epigenetic mechanisms control nucleosome spacing and how they are condensed, which subsequently determines gene activity. Briefly, chromatin exists in an inactivated and condensed state (heterochromatin) that prevents gene transcription, but when activated to an open state (euchromatin), genes can be transcribed.

It is now clear in both humans and animals that glucocorticois and stress have a significant epigenetic impact, and the relationship between the stress response and epigenetics in the brain is bidirectional (Hunter, 2012). Epigenetic alterations have become especially attractive to researchers in recent years, as increasing evidence indicates that they can be induced by physical and social exposure early in life (Meaney et al., 2007). For some neurobiological disorders, exposure to environmental agents during early developmental stages can epigenetically disturb gene regulation in a long-term manner and cause significant pathological manifestations later in life. This process is the latent early-life associated regulation model by Lahiri et al. (2009).

Epigenetic dysregulation has been associated with prenatal IUGR and disease in both humans and rodents (Baserga et al., 2007, 2010; Friso et al., 2008). Prenatal stress can cause increased DNA methylation in the frontal cortex and hippocampus (Mychasiuk et al., 2011; Matrisciano et al., 2013) and a lower DNA methyltransferase 3a immunoreactivity in the dentate gyrus in offspring (Sierksma et al., 2013).

Variations in maternal care in the rat result in differences in hippocampal development and synaptic plasticity in the offspring (Macrì and Würbel, 2006). Observational studies provide evidence for two forms of maternal behaviors during the first week of lactation: licking/grooming (LG) and the arched-back nursing (ABN) posture (Liu et al., 1997; Francis et al., 1999). In the rat, the adult offspring of high LG-ABN mothers show increased hippocampal GR expression and enhanced glucocorticoid feedback sensitivity compared to animals reared by low LG-ABN mothers (Liu et al., 1997; Francis et al., 1999). In addition, adult offspring of high LG-ABN mothers exhibited modest HPA stress responses compared to animals reared by low LG-ABN mothers (Menard and Hakvoort, 2007). In hippocampus, offspring from high LG-ABN mothers had hypomethylation of CpG dinucleotides in the exon 1_7_ GR promoter sequence, and increased histone acetylation that might account for higher transcription of the GR gene (Weaver et al., 2004). The maternal effect is mediated by enhanced serotonergic activity and an increased expression of NGFI-A, which binds the exon 1_7_ GR promoter sequence (Weaver et al., 2007). Cross-fostering experiments showed a causal relationship between maternal care and changes in the exon 1_7_ GR promoter methylation (Weaver et al., 2005, 2006).

## Epigenetic modification is a shared pathogenic substrate of both early-life stress and epilepsy

As stated above, epigenetic modifications underpin the programming effects of early-life stress. Interestingly, a wealth of evidence indicates that dysregulation of epigenetic mechanisms occurs in several human epilepsy syndromes. Epigenetic mechanisms can influence the acute deployment of genes resulting from seizures themselves or can have gradual effects on the steady-state expression profile of candidate genes that persist into epilepsy. Epigenetic modifications can affect seizure and epilepsy in several ways (Lubin, 2012; Roopra et al., 2012).

Firstly, histone acetylation is involved in epileptogenesis in human epilepsy patients. Seizure activity results in gene expression changes, including alterations in mRNA levels for glutamate receptor 2 and BDNF, the two well-characterized epileptogenesis-related genes. Of interest, histone acetyltransferase-mediated increases in histone acetylation levels at the promoter regions of the glutamate receptor 2 and BDNF genes have been shown to correlate with their gene expression changes following seizures in an experimental animal model (Huang et al., 2002b).

Secondly, DNA methylation has been highlighted as a component of the methylation hypothesis of epileptogenesis (Kobow and Blumcke, 2011). DNA methyltransferase enzymes 1 and 3a specifically, were increased in neurons from the temporal neocortices of 25 MTLE patients (Zhu et al., 2012). Using a rat model of MTLE, Williams-Karnesky et al. identified an increase in hippocampal DNA methylation that correlates with an increased DNA methyltransferase activity, disruption of adenosine homeostasis, and spontaneous recurrent seizures. To test the effects of adenosine, they used bioengineered silk implants to deliver a defined dose of adenosine over 10 days to the brains of epileptic rats (Williams-Karnesky et al., 2013). Adenosine implants reversed DNA hypermethylation seen in the epileptic brain, inhibited sprouting of mossy fibers in the hippocampus, and prevented the progression of epilepsy for at least 3 months (Williams-Karnesky et al., 2013).

Thirdly, transcription factors are involved in epileptogenesis in human epilepsy patients. Repressor element-1 silencing transcription factor and neuronal restrictive silencer factor serve to repress gene expression through dynamic recruitment of epigenetic complexes (Qureshi and Mehler, 2009). Of interest, repressor element-1 silencing transcription has been implicated in the regulation of several epileptogenesis specific factors, including growth factors, neurotransmitter receptors, ion channels, circuit excitability, and neurogenesis (Huang et al., 1999; McClelland et al., 2011a,b; Roopra et al., 2012).

Fourthly, methyl-CpG-binding protein 2 can regulate neuronal activity and is itself controlled by activity (Roopra et al., 2012).

Taken together, early-life stress can prime seizure occurrence and increases epileptogenesis. In addition, epigenetic modification is a shared pathogenic substrate of early-life stress and epilepsy.

## Coexistence of early-life stress and early-life seizures

Seizure is one of the most common pediatric emergencies, with the highest incidence in the first year of life. Animal studies have demonstrated early-life seizures differ from adult seizures by the seizure behaviors, the electroencephalogram features, and their consequences. Notwithstanding the higher susceptibility to seizures, the immature brain is less vulnerable to seizure-induced injuries than the mature brain (Dube et al., 2001; Holmes and Ben-Ari, 2001; Huang et al., 2012). However, under some circumstances seizure in the immature brain can cause permanent brain damage (Dube et al., 2006).

For humans, most early-life seizures occur in premature and sick neonates (Scher et al., 1993; Miller et al., 2002; Scher, 2003) who are hospitalized and separated from their mothers, and thus, under stress (Field, 1994; Anand, 2000). Reciprocally, early-life stress may prime the occurrence of seizures and act via glucocorticoids, thereby potentiating the excitotoxic effects of concurrent neurological insults (Sapolsky, 1996), such as seizure (Huang et al., 2002a; Lai et al., 2006).

As stated above, early-life stress can prime the seizure occurrence and subsequent epileptogenesis. Currently, more attention is being paid to the effect of early-life stress on adult-onset seizure; however, little work has focused on the effect of early-life stress on the early-life seizure (Beck and Gavin, 1976; Edwards et al., 2002; Lai et al., 2006; Young et al., 2006; Velíšek, 2011; Yum et al., 2012). Indeed, to study the coexistence of early-life stress and early-life seizures is of both experimental and clinical importance.

## The concept of development origins of health and disease (DOHaD)

Barker et al. noted that low birth weight was associated with an increased risk of adverse outcomes in adulthood, such as coronary heart disease, stroke, high blood pressure, and type 2 diabetes (Barker et al., 1989). Gluckman et al. proposed the concept of DOHaD by observing the enduring effects of the fetal environment on physical health and disease in adulthood. The process of fetal programming or developmental plasticity is one of the core assumptions of DOHaD (Gluckman et al., 2007). Gluckman et al. use the concept of predictive adaptive responses to describe the developing organism by making phenotypic responses during development to obtain an adaptive advantage (Gluckman et al., 2005). The fetus will predict and make adaptive responses to a broad range of environmental cues to aid fitness and survival in later life. If the prediction is correct, then there will be a good match between the phenotype adopted and the environment in which the organism will later live. If the prediction is poor, there will be a mismatch between the environment experienced and the phenotype induced. The authors propose that developmental mismatch triggers or exacerbates certain diseases and provide a useful explanation for the DOHaD phenomenon (Gluckman et al., 2005, 2007). Furthermore, the notion of epigenetic modifications is applied to the DOHaD approach (Waterland and Michels, 2007). The DOHaD approach has become so popular that an international society has been formed, and this society is actively promoting research and collaboration in this area.

**Figure 1:**
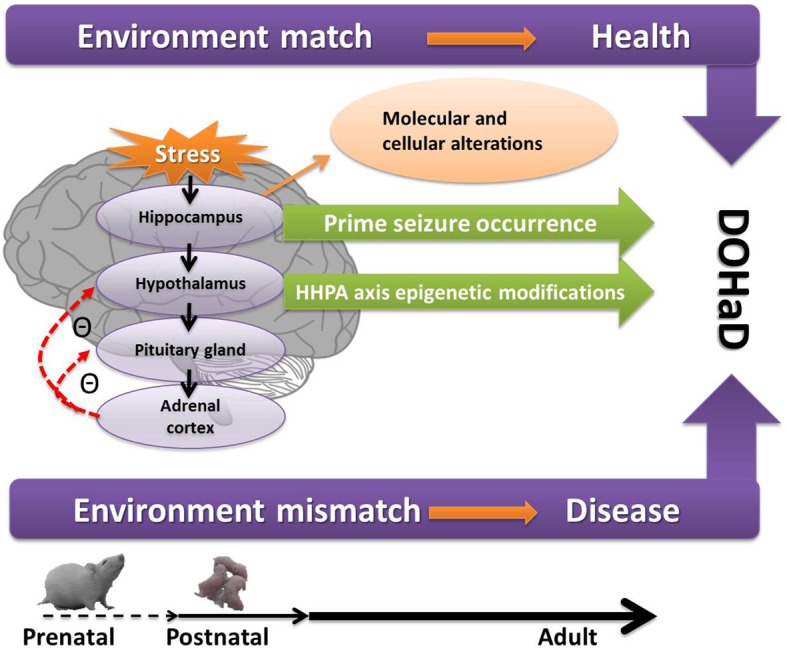
**A conceptual diagram of how prenatal and postnatal stress act on the hippocampus and HPA axis and lead to neuropsychiatric disorders through epigenetic modifications, the so-called DOHaD as presently understood.** If the early programming environment matches the later adult environment, the adults are healthy. If mismatch occurs, the adults are more likely to have diseases. Figure 1 also shows the increase seizure propensity in the context of early-life stress. See text for details. DOHaD, development origins of health and disease; HHPA, hippocampus and hypothalamic-pituitary-adrenal.

Figure 1 depicts the path form early-life adversity to long-term neuropsychiatric disorders, along with the underlying molecular and cellular mechanisms and epigenetic modifications, with a match or mismatch adaptation that leads to the final outcome.

## Conclusions and perspectives

Early-life stress can elicit detrimental effects on hippocampal development by altering the HPA axis, neuroplasticity, and behavior. Developmental plasticity allows an organism to adapt to environmental changes in the critical stages of early life. As highlighted in this review, early-life stress programs the development of the HPA axis, exerts profound effects on neural plasticity, primes seizure occurrence, and increases epileptogenesis. Epigenetic modifications play an important role in both early-life stress and epilepsy.

A number of important points made throughout the manuscript are reinforced here. Reducing damage done by prenatal and postnatal stress may help reduce the cost of treating adult diseases. Protecting pregnant mothers from harmful stress exposure and supporting programs to reduce stress or anxiety during pregnancy might lead to improvements in the health and well-being of their children later in life. Ideally, intervention and prevention should be achieved before pregnancy begins. In terms of postnatal stress, psychosocial interventions in early life can affect brain development and thereby benefit children at risk. Other perinatal adversities such as perinatal infection, nutritional disorders, and toxin exposures must be cautiously avoided and treated. The potential therapeutic value of pharmacological agents, such as CRHR_1_ antagonists, MR and GR antagonists should be explored.

Recently, an increasing number of studies have shown that early-life stress primes seizure occurrence and increases epileptogenesis. An increased understanding of the link between early-life stress and epilepsy could improve the care and treatment of patients with epilepsy, while also allowing better management of other stress-related neurological disorders.

In the future, we will need to better determine the developmental windows during which preventative or therapeutic interventions can reverse the adverse effects of developmental programming. It will also be important to better understand stress biomarkers, especially epigenetic biomarkers. An increasing number of studies have provided clues as to how early-life stress induces changes at the cellular, molecular, and epigenetic levels. Continued progress on these fronts will provide great insight into disease mechanisms, in turn leading to the potential identification of novel targets for therapy and prevention.

### Conflict of interest statement

The author declares that the research was conducted in the absence of any commercial or financial relationships that could be construed as a potential conflict of interest.
